# The Anti-inflammatory Effects of Short Chain Fatty Acids on Lipopolysaccharide- or Tumor Necrosis Factor α-Stimulated Endothelial Cells via Activation of GPR41/43 and Inhibition of HDACs

**DOI:** 10.3389/fphar.2018.00533

**Published:** 2018-05-23

**Authors:** Meng Li, Betty C. A. M. van Esch, Paul A. J. Henricks, Gert Folkerts, Johan Garssen

**Affiliations:** ^1^Division of Pharmacology, Utrecht Institute for Pharmaceutical Sciences, Faculty of Science, Utrecht University, Utrecht, Netherlands; ^2^Nutricia Research, Immunology, Utrecht, Netherlands

**Keywords:** SCFA, GPR41/43, HDACs, inflammatory cytokines, adhesion molecules

## Abstract

**Background and Aim:** Previously, we found that short chain fatty acids (SCFA) inhibit LPS or TNFα-induced endothelial inflammatory responses and excessive vascular cell adhesion molecule-1 (VCAM-1) expression, two important steps in the development of atherosclerosis. However, the mechanisms involved are still unclear. We hypothesized that the effects of SCFA are associated with activation of G-protein coupled receptor 41/43 (GPR41/43) and/or inhibition of histone deacetylases (HDACs).

**Methods:** The expression and location of GPR41/43 and HDAC3 in human umbilical vein endothelial cells (HUVEC) were confirmed. HUVEC were pre-incubated with acetate, butyrate or propionate alone or in combination with GLPG0974 (GLPG, antagonist of GPR43) or β-hydroxybutyrate (SHB, antagonist of GPR41) and then exposed to LPS or TNFα. Interleukin (IL)-6 and IL-8 levels and VCAM-1 expression were measured. HDAC activity was measured after treatment with butyrate, propionate and trichostatin A (TSA, HDAC inhibitor). The peripheral blood mononuclear cell (PBMC) adhesive level was also determined after TSA treatment.

**Results:** GPR41/43 were expressed on the membrane of HUVEC and HDAC3 was located in cytoplasm and nucleus. The GLPG and/or SHB treatments restored the inhibitory effects of acetate on IL-6 and IL-8 production and the inhibitory effects of butyrate or propionate on IL-6 production, but not on IL-8. In contrast, GLPG and/or SHB treatments did not affect the inhibitory effects of butyrate or propionate on TNFα-induced VCAM-1 expression. TSA showed similar effects on IL-8 production and VCAM-1 expression as butyrate and propionate. In addition, TSA significantly inhibited the adhesion of PBMC to an endothelial monolayer.

**Conclusion:** Activation of GPR41/43 mediates the effects of acetate on IL-6 and IL-8 production and the effects of butyrate and propionate on IL-6 production. Furthermore, inhibition of HDACs mediates the effects of butyrate and propionate on IL-8 production, VCAM-1 expression, and PBMC adhesion to an endothelial monolayer. These data indicate the beneficial roles of SCFA in preventing vascular inflammation and relevant diseases by activation of GPR41/43 and inhibition of HDACs.

## Introduction

Atherosclerosis, which is associated with chronic vascular inflammation, is an inflammatory disease with its most common pathological processes leading to cardiovascular disease (Libby, 2002). The earliest event in atherosclerosis is increased monocyte adhesion to endothelial cells, which is primarily regulated by vascular inflammatory factors including cytokines such as interleukin (IL)-6, chemokines such as IL-8 and monocyte chemoattractant protein-1, and endothelial adhesion molecules such as vascular cell adhesion molecule-1 (VCAM-1) and intracellular adhesion molecule-1 (ICAM-1) (Gwon et al., 2015). Since increases in these factors also characterize endothelial activation (Pober, 2002), regulating inflammatory reactions in the vascular endothelium is a potential target for therapeutic intervention in the treatment of atherosclerosis (Park et al., 2016).

Less endothelial activation and less low-grade inflammation have been associated, in clinical studies, with high fiber consumption (Meijer et al., 2010; van Bussel et al., 2015), which might be due to the production of short chain fatty acids (SCFA). SCFA, predominant acetate, propionate and butyrate, are the main anaerobic bacterial metabolites fermentation of dietary fibers in the colon which not only regulate proliferation, differentiation, and gene expression in the colon but also have physiological relevance for the host outside of the gastro-intestinal tract (Aoyama et al., 2010). SCFA have recently emerged as important signaling molecules regulating a variety of responses in the cardiovascular system (Richards et al., 2016). Furthermore, as we have recently shown, SCFA play beneficial roles in decreasing endothelial activation leading to diminished cytokines production and expression of adhesion molecules (Li et al., 2018). SCFA may regulate endothelial function either by inhibiting histone deacetylases (HDACs) and/or activating G-protein coupled receptors (GPRs): GPR41 (also known as free fatty acid receptor3, FFAR3) and GPR43 (or FFAR2) and/or inhibition of HDACs (Vinolo et al., 2011). However, the roles played in endothelial activation by GPRs and HDACs are not clearly understood.

GPR41 and GPR43 are differentially expressed on the membrane of cells (Sina et al., 2009; Aoyama et al., 2010; Kim et al., 2013). GPR41 is mainly expressed in blood vessel on endothelial cells, and GPR43 is mainly expressed on immune cells (Brown et al., 2003). GPR41 and GPR43 can both be activated by SCFA, but their specificities and potencies for ligands are different. The order of potency of the GPR41 is propionate ≈ butyrate > acetate and for GPR43 is propionate ≥ acetate ≈ butyrate (Bindels et al., 2013), indicating that butyrate is more active on GPR41, propionate is the most potent agonist for both GPR41 and GPR43, and acetate is more selective for GPR43 (Hong et al., 2005). Increasing evidence indicates the anti-inflammatory effects of GPR43 activation on immune cells. For example, deficiency in GPR43 increases the production of inflammatory mediators and immune cell recruitment and results in an increased inflammatory response in models of colitis, arthritis and asthma (Puddu et al., 2014). Their roles in endothelial activation remain unknown (Brown et al., 2003).

Histone deacetylases, a group of deacetylating enzymes, remove acetyl groups from both histone and non-histone proteins complexes that regulate gene expression (Delcuve et al., 2012). HDACs are involved in atherosclerosis associated inflammatory processes (Pons et al., 2009). HDAC inhibitors have shown potent anti-inflammatory activity in inflammatory diseases (Wang et al., 2007; Tang et al., 2013; Das Gupta et al., 2016). For example, HDAC3, by regulating NF-κB activity, is involved in the expression of inflammatory genes and monocyte recruitment to sites of inflammation (Leus et al., 2016). A potent HDAC inhibitor, suberoylanilide hydroxamic acid, exhibits anti-inflammatory properties by attenuating the LPS-induced expression of NF-κB-regulated cytokines (Leoni et al., 2002; Li et al., 2009). Inhibition of HDACs is also a molecular mode of action of SCFA, and butyrate and propionate but not acetate can act as HDAC inhibitors (Zheng et al., 2015). It remains unclear whether HDACs are involved in the anti-inflammatory properties of SCFA in the process of endothelial activation.

We therefore investigated the roles of GPR41/43 and HDACs in the anti-inflammatory effects of SCFA on LPS- and TNFα-induced endothelial activation. This could offer new therapeutic ways to prevent the development of atherosclerosis.

## Materials and Methods

### Reagents and Materials

Sodium butyrate, propionate, trichostatin A (TSA, a selective and reversible hydroxamate inhibitor of class I and II HDACs) (Hebbel et al., 2010), sodium β-hydroxybutyrate (SHB, antagonist for GPR41 receptor) (Kimura et al., 2011), and LPS (*Escherichia coli* 0111:B4) were purchased from Sigma-Aldrich, St. Louis, MO, United States. Sodium acetate was bought from Merck Millipore. A cytotoxicity detection kit (lactate dehydrogenase, LDH) was obtained from Roche. Human IL-6, IL-8 ELISA (enzyme-linked immunosorbent assay) kits, and calcein-AM were purchased from Invitrogen. Human recombinant TNFα, anti-human CD106 (VCAM-1) PE, and viability fixable dyes were bought from eBioscience. GLPG0974 (GLPG, antagonist of GPR43 receptor) (Namour et al., 2016) was obtained from Tocris Bioscience. Primary anti-GPR41 antibody, anti-GPR43 antibody, and an HDAC activity assay kit were purchased from Abcam. EGM-2 Bulletkit was purchased from Lonza (Switzerland).

### Cell Culture

Human umbilical vein endothelial cells (HUVEC) from umbilical vein were provided by Mrs. J. H. van Kats-Renaud (University Medical Center, Utrecht). HUVEC were isolated and cultured by adapting the method of Jaffe et al. (1973). HUVEC were cultured in EGM-2 (Lonza) containing 2% fetal bovine serum (FBS) and VEGF for rapid proliferation in a humidified incubator at 37°C in 5% CO_2_ and medium was changed every 2–3 days. Cells of passage 2–7 were used. Informed consent was obtained from all subjects and was provided in accordance with the Declaration of Helsinki. Approval was obtained from the Medical Ethics Committee of the University Medical Center Utrecht (Utrecht, Netherlands).

### PBMC Isolation

Human peripheral blood mononuclear cell (PBMC) from healthy donors were isolated from buffy coats (Sanquin, Amsterdam, Netherlands). Cells were purified using Ficoll-Paque PLUS gradient centrifugation (de Kivit et al., 2011). Briefly, PBMC were isolated by centrifugation according to the manufacturer’s instructions. PBMC above the polyester gel were collected, washed with PBS containing 2% FBS by centrifuging at 1000 *g* for 13 min, re-suspended cell pellet at a concentration of 2 × 10^6^ cells/ml in RPMI1640 medium without phenol red containing 10% FBS and 1% penicillin-streptomycin. The viability of PBMC was determined by trypan blue staining and cell number was counted. Informed consent was obtained from all subjects and was provided in accordance with the Declaration of Helsinki.

### Cell Cytotoxicity (LDH) Test

Based on the results published in the recent manuscript (Li et al., 2018), we chose different exposure periods for each SCFA in the present study. HUVEC were treated with acetate (10 mM), TSA (1 μM), SHB (5 mM), and GLPG (0.1 μM) alone or in combination for 28 h. Treatment with propionate (0.3 mM) and butyrate (0.1 mM), alone or in combination with antagonists or TSA lasted for 48 h. Treatment with propionate (10 mM) and butyrate (5 mM), alone or in combination with antagonists or TSA lasted for 32 h. After treatments, supernatants were transferred into a new 96-well plate and 100 μl reaction solution of LDH was added into each well. The plate was incubated in the dark at room temperature for 30 min and was then measured at 490 nm excitation and 650 nm emission by microplate reader. Positive control was 1% Triton-X treated cells, which led to 100% cell death.

### Immunocytochemistry

Human umbilical vein endothelial cells (5 × 10^3^ cells) were seeded to 96-well plate and incubated at 37°C, 5% CO_2_ incubator for two days. The cells then were processed for immunocytochemistry as previously described (Westenbroek et al., 2013). Briefly, the HUVEC were washed twice by cold PBS. For intracellularly located protein (HDAC3), cells were treated with permeabilization solution (0.25% Triton-X) for 10 min and then washed with cold PBS. HUVEC were then incubated with blocking buffer for 1 h and washed with cold PBS. Cells were subsequently stained with primary antibodies, rabbit-anti-human GPR41 (1:300), rabbit-anti-human GRP43 (1:300), and rabbit-anti-human HDAC3 antibodies (1:800), overnight at 4°C. HUVEC were then incubated with goat anti-rabbit Alexa Fluor 488 or 568 secondary antibodies (1:400) for 1 h and washed with cold PBS three to five times. Negative controls were simply stained with secondary antibodies.

### ELISA for Pro-inflammatory Cytokines Detection

Confluent HUVEC were treated with acetate (10 mM) alone or in combination with GLPG (0.1 μM) or SHB (5 mM) for 16 h and were then exposed to LPS (1 μg/mL) or TNFα (1 ng/mL) for 12 h. Propionate (0.3 mM) and butyrate (0.1 mM) alone or in combination with GLPG or SHB were treated for 24 h, then stimulated with LPS or TNFα for 24 h. Supernatants were collected and stored at -20°C. The levels of inflammatory cytokines IL-6 and IL-8 were assayed by ELISA according to the manufacturer’s instructions using standard curve. The optical densities of the samples were detected using a microplate reader at a wavelength of 450 nm.

### HDAC Activity Assay

After treatment with propionate (0.3 mM/10 mM) and butyrate (0.1 mM/5 mM) for 3, 6, 12 or 48 h, cells lysates were collected and stored in -80°C until measurement. HDAC activity was detected with flurometric HDAC activity assay kit according to manufacturer’s protocol. As a positive control, the effects of TSA (0.01μM, 0.1μM and 1μM) on HDAC activity were also measured after 48 h treatment. Fluorescence was monitored every 1 min over 60 min at 37°C using a Fluostar reader at excitation wavelength of 355 nm and emission wavelength of 460 nm.

### Flow Cytometry

Confluent HUVEC were incubated with propionate (10 mM) and butyrate (5 mM) alone or in combination with GLPG, SHB or TSA for 24 h. HUVEC were then stimulated with TNFα (1 ng/mL) for 8 h. After treatment, HUVEC were stained with human VCAM-1 PE-conjugated antibody and cell viability dye according to the manufacturer’s protocol and then detached from the culture plates with 0.05% trypsin-EDTA. The stained HUVEC were measured by flow cytometer FACSCanto II and data was analyzed by Flowlogic version7.

### Endothelial Cell Monolayer Adhesion Experiment

Human umbilical vein endothelial cells were seeded in 96-well plates until they were confluent. PBMC, isolated from different healthy donors, were washed with warm PBS three times, and PBMC (2 × 10^6^/mL) labeled with 1 μM calcein-AM for 30 min according to the manufacturer’s instruction. HUVEC were pre-incubated with TSA (1 μM) for 24 h, followed by 8 h of TNFα stimulation. After stimulation, the medium was exchanged for fresh medium and co-cultured with labeled PBMC (2 × 10^5^ cells/well) for 30 min. The un-adhesive PBMC were then washed away and HUVEC with adhesive PBMC were fixed with 4% paraformaldehyde. The fluorescence image of labeled PBMC was captured by Yokogawa CV7000S imager and relative fluorescence intensity was measured by fluoroskan Ascent^TM^ FL with excitation wavelength 492 nm and emission wavelength 518 nm.

### Statistical Analysis

All data are expressed as mean ± standard deviation (SD). Group comparisons were performed using the one-way ANOVA analysis of variance of the experiments. The method of least-significant difference (LSD) was used as a *post hoc* test for multiple comparisons, to determine significant difference between specific treatment groups. In all cases, *P-value* < 0.05 was considered statistically significant.

## Results

### No Compounds Used Showed Cytotoxicity

Human umbilical vein endothelial cells were treated with different concentrations of SHB, GLPG and TSA to test cytotoxicity (**Supplementary Figures S1A–C**). Non-toxic concentrations of SHB (5 mM), GLPG (0.1 μM), and TSA (1 μM) were used in the following experiments.

Human umbilical vein endothelial cells were treated for 28 h or 48 h with acetate (10 mM), propionate (0.3 mM), and butyrate (0.1 mM) in combination with SHB (5 mM), GLPG (0.1 μM), or TSA (1 μM) (**Supplementary Figures S1D–F**), while propionate (10 mM) and butyrate (5 mM) combinations were treated for 32 h (**Supplementary Figures S1G,H**). Medium was collected for the measurement of LDH release, an indicator of cytotoxicity. All the treatments with acetate, propionate or butyrate in combination with GLPG, TSA and SHB were non-toxic.

### GPR41 and GPR43 Was Expressed on the HUVEC Membrane

GPR41 and GPR43 are the two targets of SCFA which have been indicated to modulate the inflammation and immune responses in non-endothelial cells (Ang and Ding, 2016; Corrêa-Oliveira et al., 2016). To ascertain the expression of GPR41 and GPR43 on the HUVEC membrane, HUVEC were stained with GPR41 and GPR43 antibodies and checked with a Yokogawa CV7000S imager. GPR41 (**Supplementary Figure S2C**) and GPR43 (**Supplementary Figure S2D**) were expressed on HUVEC. Cells stained only with the secondary antibodies were regarded as backgrounds (**Supplementary Figures S2A,B**).

### HDAC3 Was Located in the Cytoplasm and Nucleus

Histone deacetylases activity can be inhibited by SCFA, especially butyrate and propionate, and regulate gene expression mediating the effects of SCFA (Vinolo et al., 2011; Corrêa-Oliveira et al., 2016). HDACs, including HDAC3, a class I HDAC, are highly expressed in endothelial cells (Lee et al., 2012). We confirmed that HDAC3 was expressed in HUVEC, and that HDAC3 was highly expressed in the cytoplasm and nucleus (**Supplementary Figures S2E**).

### Acetate Inhibited Pro-inflammatory Cytokine (IL-6 and IL-8) Production in LPS- or TNFα-Stimulated HUVEC via Activation of GPR41 and GPR43

To determine whether GPR41/43 are involved in the decrease of IL-6 and IL-8 production by acetate, we compared the effects of acetate alone or in combination with antagonists of GPR41/43. Optimum concentrations and incubation times for SCFA and LPS/TNFα were determined previously (Li et al., 2018). Acetate treatment alone showed no significant effects on IL-6 and IL-8 production compared to the control group (data not shown).

#### Acetate-Inhibited IL-6 Production Was Associated With Activation of GPR41/43

**FIGURE 1:**
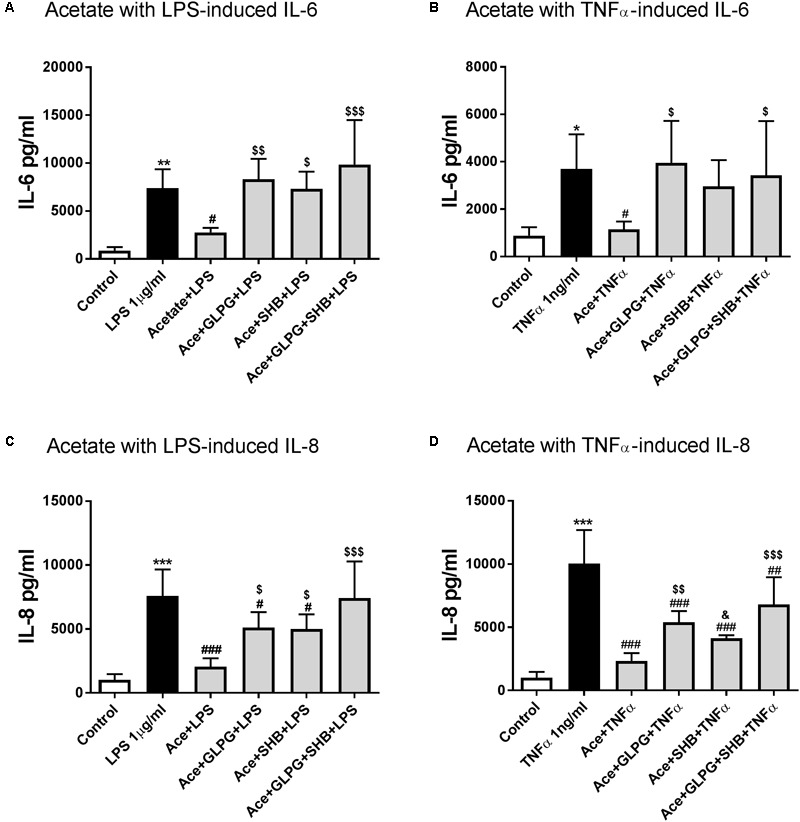
The effects of acetate alone or in combination with GLPG and/or SHB on IL-6 and IL-8 production by LPS- or TNFα-stimulated HUVEC. **(A)** and **(B)** show the effects of pre-treatment with acetate alone or combined with GLPG and/or SHB for 16 h on IL-6 production stimulated by LPS or TNFα for 12 h; **(C)** and **(D)** show the IL-8 production level after pre-incubating acetate or in combination with GLPG and/or SHB for 16 h and 12 h LPS or TNFα stimulation. *N* = 4, ^∗^*p* < 0.05, ^∗∗^*p* < 0.01, ^∗∗∗^*p* < 0.001 compared with control group; ^#^*p* < 0.05, ^###^*p* < 0.001 compared with LPS or TNFα group; ^$^*p* < 0.05, ^$$^*p* < 0.01, ^$$$^*p* < 0.001 compared with acetate alone group; ^&^*p* < 0.05 compared with acetate in combination with GLPG/SHB (AGS) group.

LPS (1 μg/ml for 12 h) significantly enhanced the IL-6 production of HUVEC (**Figure 1A**). Similar results were observed for TNFα (1 ng/ml, **Figure 1B**). IL-6 production was significantly reduced by pre-incubation with acetate (10 mM for 16 h), and was completely restored by the GPR41 or GPR43 antagonist (SHB or GLPG) alone or in combination (**Figures 1A,B**).

Furthermore, in the acetate and GPR41/43 antagonists combination group, IL-6 production showed no significant difference between acetate combined with GPR41 or GPR43 antagonist alone (**Figure 1A**). Similar effects of acetate on TNFα-induced IL-6 production were observed; however, treatment with acetate in combination with GPR41 antagonist (SHB) partially increased IL-6 production (**Figure 1B**). These data indicate that both GPR41 and GPR43 are involved in the inhibitory effects of acetate on IL-6 production.

#### Acetate-Inhibited IL-8 Production Was Associated With Activation of GPR41/43

After pre-incubation with acetate for 16 h, the IL-8 release was significantly decreased in LPS stimulated group (12 h) (**Figure 1C**). IL-8 levels were partially restored by GLPG (GPR43 antagonist) or SHB (GPR41 antagonist), but still lower than the LPS stimulation group (**Figure 1C**). However, IL-8 production was completely restored in the GLPG/SHB-treated group (**Figure 1C**). In addition, acetate in combination with both GPR41 and GPR43 antagonist showed additional effects on IL-8 production compared with each antagonist alone (**Figure 1C**). Results suggest that the effects of acetate on IL-8 production are mediated by both by GPR41 and GPR43. In the TNFα-treated groups, IL-8 production was significantly reduced by acetate, an effect that was significantly inhibited by treatment with GPR43 antagonist and partially inhibited by GPR41 antagonist (**Figure 1D**). However, this decrease in TNFα-induced IL-8 production was not completely restored by GPR41/43 antagonist treatment, which led to levels still significantly lower than those of the LPS-stimulated group. These results together indicate that both GPR41 and GPR43 are involved in the effects of acetate on TNFα-induced IL-8 production.

### Butyrate and Propionate Inhibited IL-6 Production Mainly via Activation of GPR41/43

**FIGURE 2:**
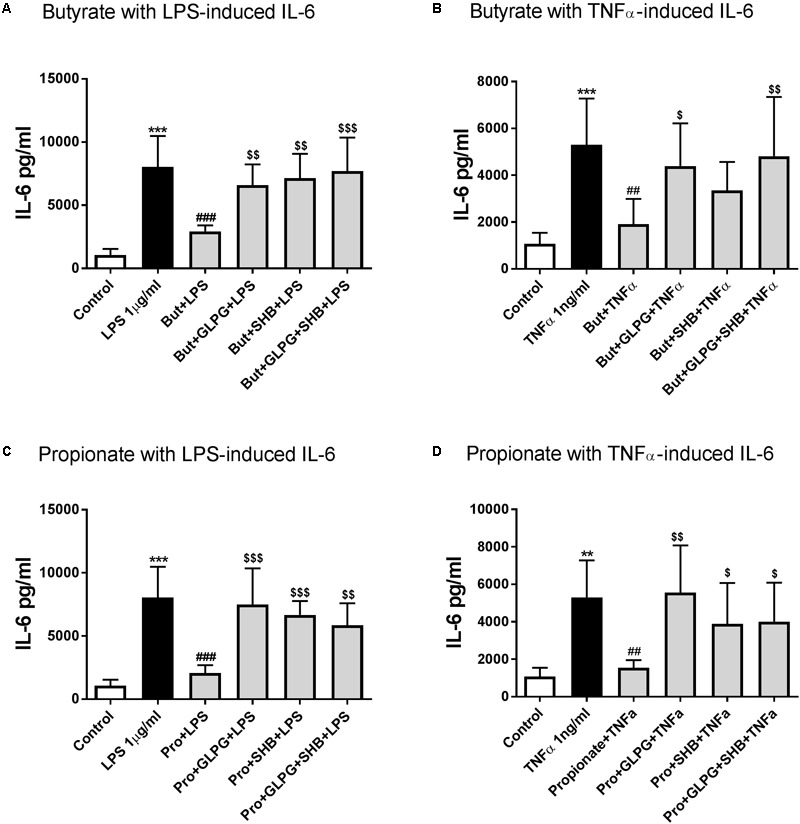
Butyrate and propionate inhibited IL-6 in LPS and TNFα-stimulated HUVEC. Pre-treatment with butyrate (0.1 mM) and propionate (0.3 mM) for 24 h significantly reduced IL-6 levels in 12 h LPS- or TNFα-stimulated groups. **(A)** and **(B)** show the effects of butyrate; **(C)** and **(D)** show the role of GLPG and/or SHB on the effects of propionate; *N* = 4, ^∗∗^*p* < 0.01, ^∗∗∗^*p* < 0.001 compared with control group; ^##^*p* < 0.01, ^###^*p* < 0.001 compared with LPS or TNFα group; ^$^*p* < 0.05, ^$$^*p* < 0.01, ^$$$^*p* < 0.001 compared with butyrate or propionate alone group.

Butyrate or propionate treatment alone showed no significant effects on IL-6 production compared to the control group (data not shown). IL-6 production was significantly decreased by pre-treatment with butyrate for 24 h in the LPS-stimulated (12 h) group. The effect of butyrate treatment alone was reversed when combined with GLPG or SHB (**Figure 2A**). Similar effects of butyrate in combination with GLPG were found in TNFα-activated groups (**Figure 2B**). However, the effect of butyrate on IL-6 production was only partially reversed by SHB treatment but not significantly different from the TNFα stimulated group (**Figure 2B**). IL-6 production was also inhibited by propionate treatment (**Figures 2C,D**). Again, this was significantly reversed when combined with GLPG or SHB (**Figures 2C,D**).

### HDACs Are Involved in Butyrate and Propionate-Reduced IL-8 Production in LPS or TNFα-Stimulated HUVEC

**FIGURE 3:**
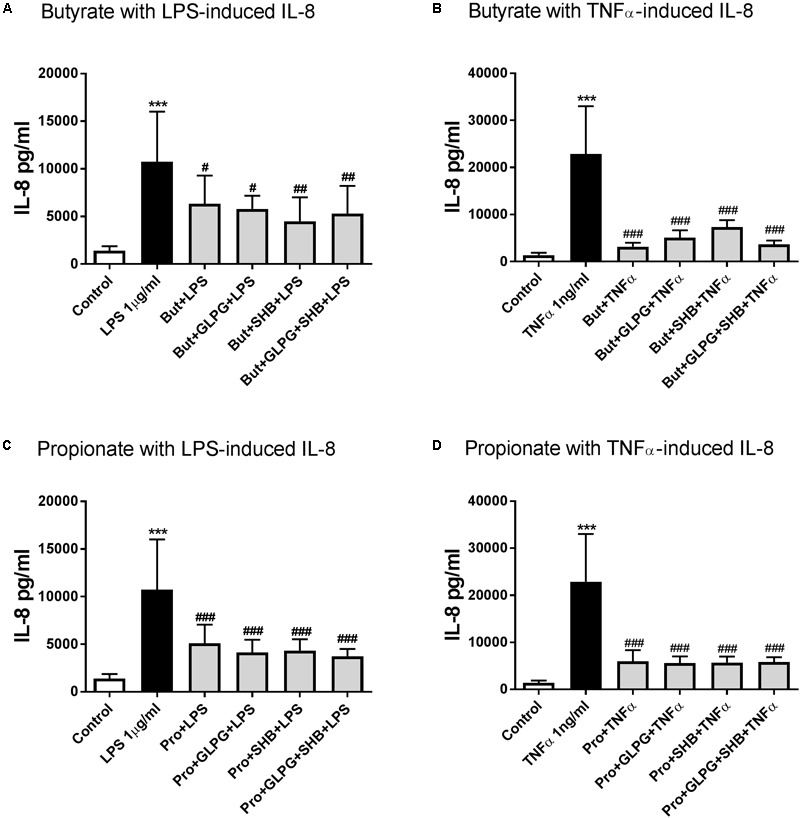
Butyrate and propionate inhibited IL-8 production in LPS and TNFα-stimulated HUVEC. With 24 h pre-treatment, butyrate and propionate reduced IL-8 production in 24 h LPS or TNFα stimulation. **(A)** and **(B)** are the data of butyrate effects on LPS- and TNFα-induced IL-8 production, respectively; **(C)** and **(D)** are the data of propionate treatment. *N* = 4, ^∗∗∗^*p* < 0.001 compared with control group; ^#^*p* < 0.05, ^##^*p* < 0.01, ^###^*p* < 0.001 compared with LPS or TNFα group.

Butyrate or propionate treatment alone showed no significant effects on IL-8 production compared to the control group (data not shown). In both the 24 h LPS and TNFα-activated groups, IL-8 production was significantly decreased by 24 h pre-treatment with butyrate. These effects were not altered by GLPG or SHB (**Figures 3A,B**). Similar effects on IL-8 production were shown by treatment with propionate production (**Figures 3C,D**). Therefore, we hypothesized that HDACs might be involved in the regulation of IL-8.

**FIGURE 4:**
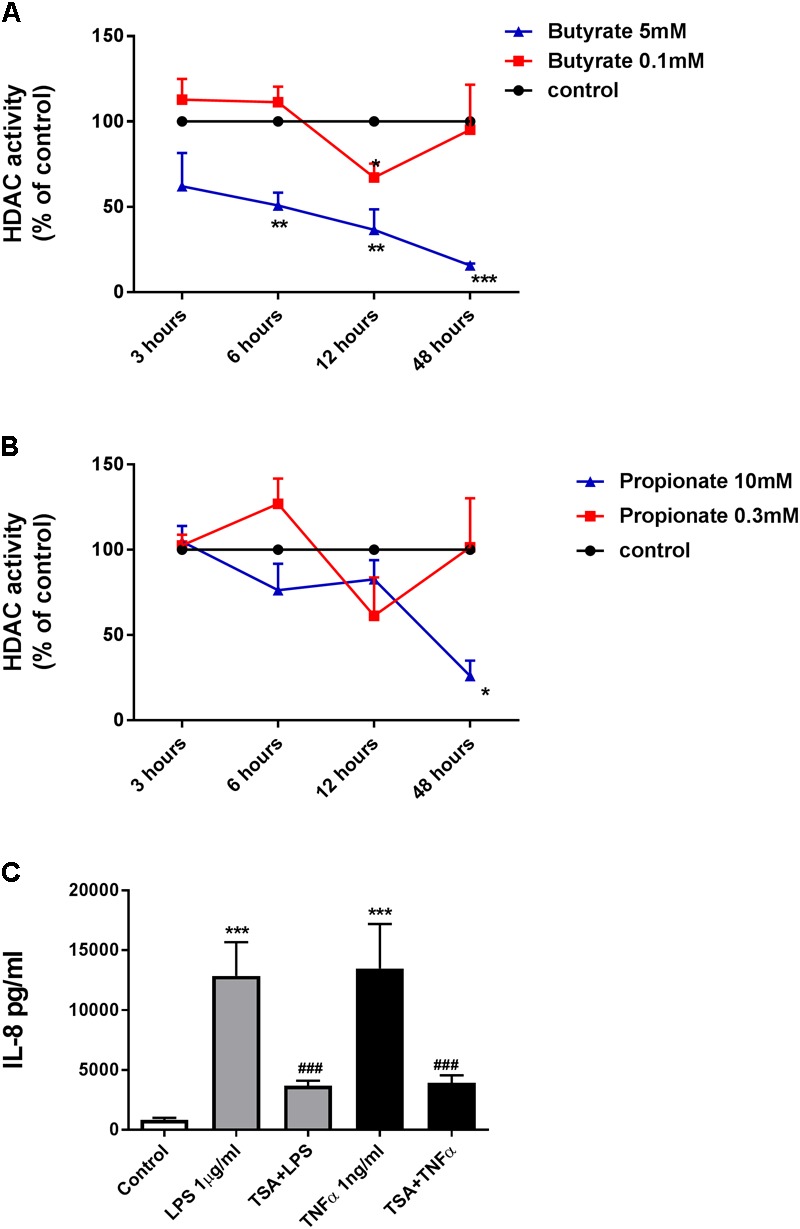
Butyrate, propionate, and TSA inhibited HDACs activity in HUVEC. **(A)** and **(B)** HDACs activity were inhibited in HUVEC by treatment of butyrate and propionate. The results were normalized using the control as 100%. **(C)** TSA inhibited IL-8 production in HUVEC. *N* = 3–4 for each group, ^∗^*p* < 0.05, ^∗∗^*p* < 0.01, ^∗∗∗^*p* < 0.001 compared with control group; ^###^*p* < 0.001 compared with LPS or TNFα group.

For this reason, we measured the effects of butyrate and propionate on HDAC activity with different concentrations (butyrate: 0.1 mM and 5 mM; propionate: 0.3 mM and 10 mM) within 48 h. Results indicated that butyrate (0.1 mM) and propionate (0.3 mM) inhibited HDAC activity after 12 h treatment. Butyrate (5 mM) significantly inhibited HDAC activity after 6 h treatment, while the inhibitory effect of propionate (10 mM) on HDAC activity reached significance after 48 h treatment (**Figures 4A,B**). TSA, as a positive control, showed a concentration-dependent inhibitory effect on HDAC activity (**Supplementary Figure S3**). Moreover, inhibitory effects on IL-8 production were also shown by the HDAC inhibitor TSA (**Figure 4C**). These data indicate that HDACs might be involved in the effects of butyrate and propionate on IL-8 production.

### Butyrate and Propionate Suppression of VCAM-1 in TNFα-Stimulated HUVEC Were Associated With Inhibition of HDACs but Not With Activation of GPR41/43

**FIGURE 5:**
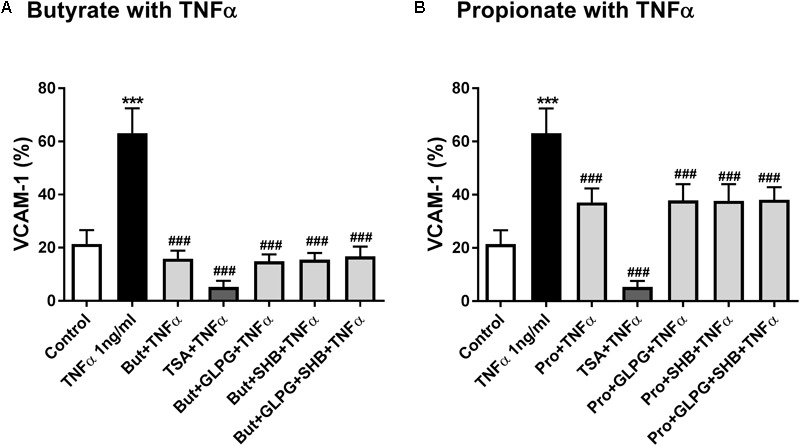
Butyrate and propionate inhibited TNFα-induced VCAM-1 expression. HUVEC were pre-treated with propionate (10 mM), butyrate (5 mM), GLPG (0.1 μM), SHB (5 mM), TSA (1 μM), or their combinations and then exposed to TNFα (1 ng/mL). **(A)** Shows the effects of butyrate or its combination with GLPG and/or SHB on VCAM-1 expression; **(B)** shows the effects of propionate or its combination with GLPG and/or SHB on VCAM-1 expression. *N* = 5. ^∗∗∗^*p* < 0.001 compared with control group; ^###^*p* < 0.001 compared with TNFα group.

Based on previous experiments (Li et al., 2018), propionate (10 mM), butyrate (5 mM), and TSA (1 μM), either alone or in combination with antagonists, were used to pretreat confluent HUVEC for 24 h and then exposed to TNFα (1 ng/mL) for 8 h. TNFα increased the VCAM-1 expression (**Figure 5**). Butyrate, propionate and TSA all inhibited TNFα-induced VCAM-1 expression, and the effect was not reversed by GLPG and/or SHB treatment (**Figures 5A,B**). These results indicate that, unlike GPR41 and GPR43, HDACs were involved in the inhibitory effects of butyrate and propionate on TNFα-induced VCAM-1 expression.

### TSA Inhibited Adhesion of PBMC to Endothelial Cells

**FIGURE 6:**
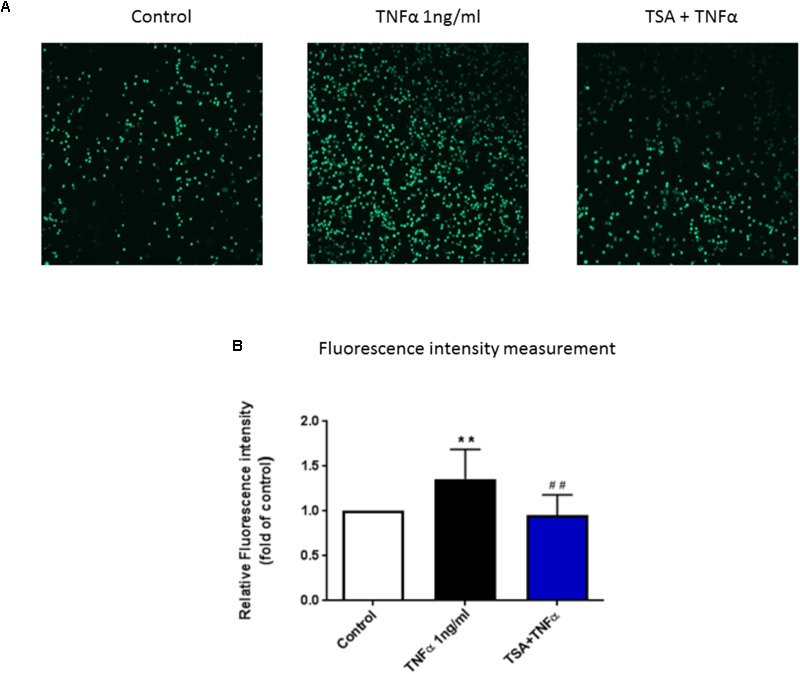
The functional role of TSA on PBMC adhesion to HUVEC monolayer. Until HUVEC were confluent, cells were pre-treated with TSA (1 μM) for 24 h, followed by 8 h TNFα stimulation, then co-cultured with calcein-AM labeled PBMC for 30 min. **(A)** Shows adhesive PBMC visualized by Yokogawa CV7000S imager; **(B)** shows the mean data of relative fluorescence intensity of adhesive PBMC presented as the fold of control. *N* = 8. ^∗∗^*p* < 0.01 compared with control group; ^##^*p* < 0.01 compared with TNFα group.

To investigate the functional role of TSA on PBMC adhesion to vascular endothelium, we conducted an adhesion assay by co-culturing PBMC and HUVEC. The adhesion of PBMC to HUVEC was significantly increased by TNFα (1 ng/ml). In a previous study, we showed that butyrate and propionate inhibited the adhesion of activated mononuclear cells to stimulated HUVEC (Li et al., 2018). In the current study we also observed a significant inhibition by TSA (**Figure 6**).

## Discussion

Excessive pro-inflammatory cytokines production and adhesive molecule expression are two important steps in the development of atherosclerosis (Gwon et al., 2015). We have previously demonstrated that SCFA pre-treatment could significantly inhibit IL-6 and IL-8 production as well as VCAM-1 expression on activated endothelial cells (Li et al., 2018). However, the mechanisms involved in their anti-inflammatory effects remained unclear and an understanding of their mechanisms of action could be important for new therapeutic options for atherosclerosis and for related lifestyle adjustments. We confirmed the membrane expression of GPR41/43 and the intracellular location of HDAC3 on HUVEC, which indicate that the anti-inflammatory effects of SCFA on activated endothelial cells might be mediated by activation of GPR41/43 and/or inhibition of HDACs. Furthermore, we found that GPR41/43 are involved in cytokine production but not in adhesion molecule expression and that HDACs are involved in the butyrate and propionate-induced decrease in IL-8 production and VCAM-1 expression.

Inflammatory mediators, such as IL-6 and IL-8, are crucial to the pathophysiology of atherosclerosis (Agarwal et al., 2013; Bi et al., 2016). IL-6 is an important upstream inflammatory cytokine in propagating the downstream inflammatory response for atherosclerosis (Hartman and Frishman, 2014). IL-8 is a prominent chemokine that triggers the adhesion, transmigration and retention of monocytes and macrophages to the atherosclerotic sites (Boisvert, 2004). Therefore, decreasing IL-6 and IL-8 should be an important step in preventing the development of atherosclerosis. In the present study, acetate reduced IL-6 and IL-8 production in LPS- or TNFα-activated HUVEC, which were prevented by co-treatment with GPR41 or GPR43 antagonist. Furthermore, the effects of GPR43 antagonist seem more profound than those of the GPR41 antagonist, which corresponds with the activation potency order of acetate on GPR41 and GPR43 (Bindels et al., 2013). Butyrate and propionate have effects on IL-6 production similar to those of acetate, but IL-8 production by stimulated HUVEC was unchanged by GPR41 or GPR43 antagonist treatments, suggesting that the inhibitory effects of butyrate and propionate on IL-8 production were not mediated by activation of GPR41/43. IL-8 is encoded on the CXCL8 gene, which is normally repressed due to histone deacetylation (Jundi and Greene, 2015), thus theoretically, inhibition of histone deacetylase results in hyper-acetylation of histones and, increases IL-8 production. However, in our study, butyrate and propionate inhibited IL-8 production and HDAC activity. Furthermore, TSA, as a potent HDACs inhibitor, also reduced IL-8 production. These findings are in agreement with previous studies that show a reduction in IL-8 gene expression by butyrate but accompanied with induced histone H4 hyper-acetylation in its inducible promoter (Huang et al., 1997; Hoshimoto et al., 2002). Taken together, these results indicate that HDACs might be involved in the effects of butyrate and propionate on the IL-8 production.

In addition to the cytokines and chemokines, monocyte migration and adhesion to the inflammation site on endothelial cells is another essential cellular event for initiation of inflammatory processes associated with atherosclerosis (Bozic et al., 2015). Cell adhesion is a multi-step process including rolling, firm adhesion, and transmigration of the endothelial monolayer and is regulated by a combination of endothelial cell surface adhesion molecules including VCAM-1, ICAM-1, and selectins (Burris et al., 2014; Park et al., 2016). Furthermore, deletion of VCAM-1 expression has been shown to suppress atherosclerotic lesions in hyper-lipidemic mouse models (Burris et al., 2014). This shows the importance of VCAM-1 in cell adhesion and the development of atherosclerosis. VCAM-1, also known as CD106, is extensively expressed on activated endothelial cells and mediates both rolling-type adhesion and firm adhesion steps during monocyte adhesion and transmigration (Burris et al., 2014; Vogel et al., 2015). Therefore, decreasing VCAM-1 expression might be beneficial in inhibiting inflammation and the development of atherosclerosis. In a recent study, we showed that VCAM-1 expression is inhibited by butyrate and propionate but not by acetate (Li et al., 2018).

VCAM-1 expression in TNFα stimulated endothelial cells has been shown to be regulated by the acetylation status and TSA increased intracellular acetylation leading to significantly suppress TNFα-induced VCAM-1 expression in *in vitro* and *in vivo* experiments (Inoue et al., 2006; Park et al., 2016). Furthermore, siRNA knockdown of HDAC3 in HUVEC reduced VCAM-1 expression and hence suppressed monocyte adhesion (Cantley and Haynes, 2013). Such data demonstrates the involvement of HDACs, especially HDAC3, in regulating VCAM-1 expression. We found that acetate, which has no HDAC inhibitory activity (Waldecker et al., 2008), showed no effect on TNFα-induced VCAM-1 expression (Li et al., 2018). Moreover, activation of GPR41/GPR43 did not mediate the suppression of VCAM-1 expression by butyrate and propionate. Finally, TSA, a potent HDAC inhibitor, significantly inhibited VCAM-1 expression, and this effect was more potent than butyrate and propionate. Furthermore, the PBMC adhesion level was significantly inhibited by TSA which showed effects similar to those of butyrate and propionate (Li et al., 2018). Based on our data, we conclude that the effects of butyrate and propionate on VCAM-1 expression were probably mediated by inhibition of HDACs thereby inhibiting the subsequent immune cell adhesion and preventing the development of atherosclerosis.

The present study offers novel insights into the anti-inflammatory mechanisms of SCFA in stimulated human primary endothelial cells and shows the different involvement of GPR41/43 and HDACs in the anti-inflammatory process of SCFA including inhibition of the pro-inflammatory cytokines production and down-regulation of adhesion molecule expression on HUVEC. We found that both activation of GPR41/43 and inhibition of HDACs are involved in decreasing pro-inflammatory cytokines production, IL-6 and IL-8, in LPS- and TNFα-activated HUVEC. Inhibition of HDACs, and not activation of GPR41/43, significantly attenuated VCAM-1 expression in TNFα-activated HUVEC and consequently inhibited PBMC adhesion to endothelial monolayer. Thus, modulating GPR41/43 and/or HDACs might be a promising therapeutic pathway for the treatment of vascular inflammation relevant diseases, including atherosclerosis. Moreover, these findings argue for altering lifestyles in the direction of increased dietary intake of fibers that promote the production of SCFA.

## Author Contributions

ML performed the experiments and analyzed the data and wrote the article. All authors were responsible for the study design and the interpretation of the data, helped to write the manuscript, and approved the final version.

## Conflict of Interest Statement

Authors BvE and JG were employed by company Nutricia Research. All other authors declare no competing interests. The abovementioned company had no role in study design, data collection and analysis, decision to publish or preparation of the manuscript.

## Supplementary Material

FIGURE S1 LDH cell toxicity test. Toxic effects of **(A)** GLPG, **(B)** TSA, **(C)** SHB, **(D)** acetate (10 mM) combination, **(E)** butyrate (0.1 mM) combination, **(F)** propionate (0.3 mM) combination, **(G)** butyrate (5 mM) combination, and **(H)** propionate (10 mM) combination. Cells treated only with medium were control groups and cells exposed to 1% Triton-X were used as a positive control of cytotoxicity. *N* = 5–6, ^∗∗∗^*p* < 0.001 compared with control group.

FIGURE S2 GPR41 and GPR43 were expressed on HUVEC membrane. Only staining with secondary antibodies **(A,B)** was regarded as background staining. HUVEC were stained with **(C)** rabbit anti-human GPR41 and **(D)** GPR43 antibodies, then stained with goat anti-rabbit Alexa Fluor 488 (green) or 568 (orange) second antibodies. HDAC3 was located in cytoplasm and nucleus in HUVEC **(E)**. HUVEC were fixed and permeabilized by 1% paraformaldehyde and 0.25% Triton-X. Fixed cells were stained with HDAC3 primary antibody and Alexa Fluor 568 (orange) secondary antibody.

FIGURE S3 TSA inhibited HDAC activity in HUVEC. TSA concentration-dependently inhibited HDAC activity in HUVEC at 48 h. The results were normalized using the control as 100%. *N* = 4.
