# Supplementary figures and images for: The Anti-inflammatory Effects of Short Chain Fatty Acids on Lipopolysaccharide- or Tumor Necrosis Factor α-Stimulated Endothelial Cells via Activation of GPR41/43 and Inhibition of HDACs
Source: Front Pharmacol. 2018 May 23;9:533. doi: 10.3389/fphar.2018.00533 (PMC5974203; doi:10.3389/fphar.2018.00533)

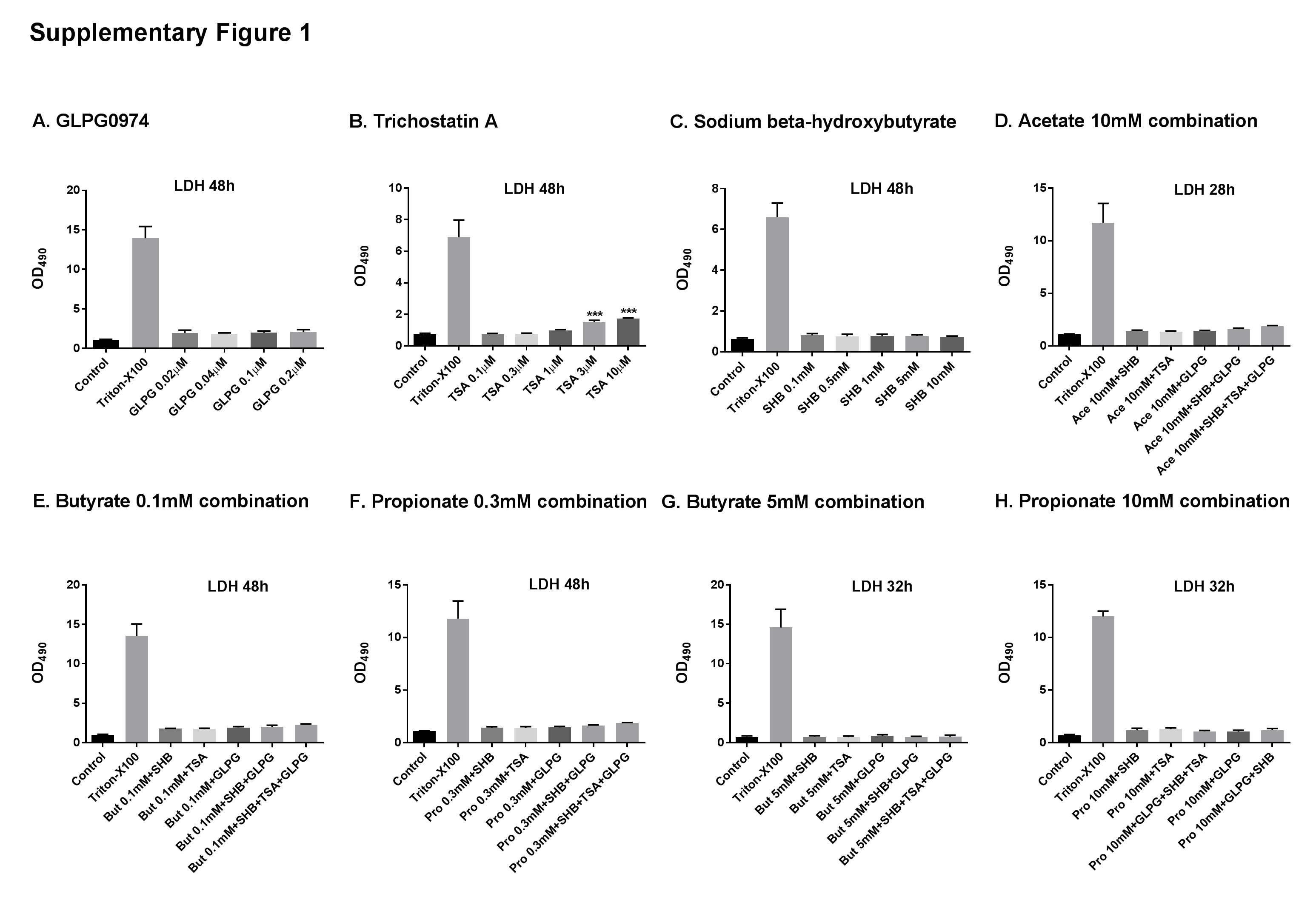

Supplement: FIGURE S1 — LDH cell toxicity test. Toxic effects of (A) GLPG, (B) TSA, (C) SHB, (D) acetate (10 mM) combination, (E) butyrate (0.1 mM) combination, (F) propionate (0.3 mM) combination, (G) butyrate (5 mM) combination, and (H) propionate (10 mM) combination. Cells treated only with medium were control groups and cells exposed to 1% Triton-X were used as a positive control of cytotoxicity. N = 5–6, ∗∗∗p < 0.001 compared with control group. [file Image_1.TIF]

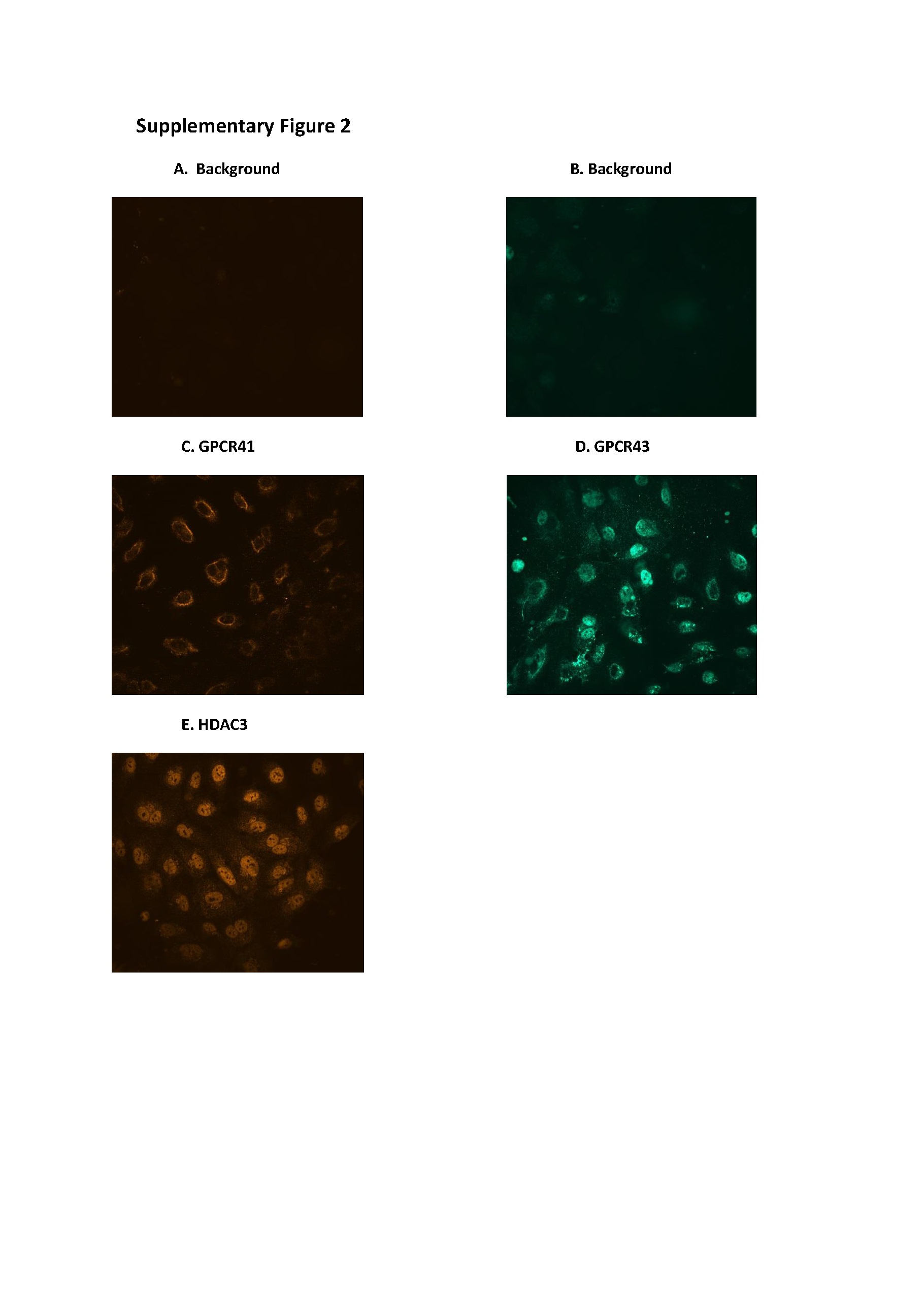

Supplement: FIGURE S2 — GPR41 and GPR43 were expressed on HUVEC membrane. Only staining with secondary antibodies (A,B) was regarded as background staining. HUVEC were stained with (C) rabbit anti-human GPR41 and (D) GPR43 antibodies, then stained with goat anti-rabbit Alexa Fluor 488 (green) or 568 (orange) second antibodies. HDAC3 was located in cytoplasm and nucleus in HUVEC (E). HUVEC were fixed and permeabilized by 1% paraformaldehyde and 0.25% Triton-X. Fixed cells were stained with HDAC3 primary antibody and Alexa Fluor 568 (orange) secondary antibody. [file Image_2.TIF]

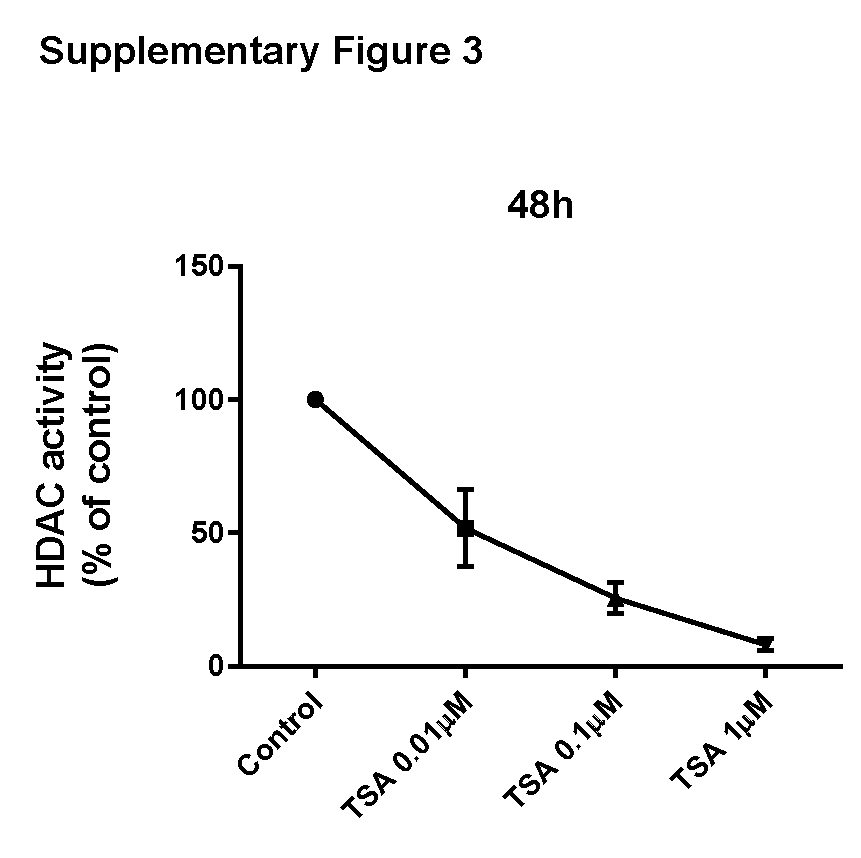

Supplement: FIGURE S3 — TSA inhibited HDAC activity in HUVEC. TSA concentration-dependently inhibited HDAC activity in HUVEC at 48 h. The results were normalized using the control as 100%. N = 4. [file Image_3.TIF]
